# A Retrospective Study on Spinal Dissemination of Supratentorial Glioma

**DOI:** 10.3389/fonc.2021.765399

**Published:** 2021-12-22

**Authors:** Jianxin Chen, Fan Yang, Qi Shi, Yuze Zhao, Hongyan Huang

**Affiliations:** Department of Oncology, Beijing Shijitan Hospital, Capital Medical University, Beijing, China

**Keywords:** supratentorial glioma, metastatic spinal dissemination (MSD), overall survival (OS), prognosis, chemotherapy

## Abstract

**Objective:**

Metastatic spinal dissemination (MSD) of supratentorial glioma is very rare and there is no established standard of care. The current study investigates the clinical characteristics and course of spinal dissemination of supratentorial glioma.

**Methods:**

A retrospective analysis of adult patients with MSD of supratentorial glioma treated in the Department of Oncology in Beijing Shijitan Hospital, Capital Medical University from June 2012 until August 2021 was performed. The time to event was estimated using Kaplan–Meier analysis. Univariate analyses were performed using log-rank test and multivariate analysis was performed using the Cox proportional hazards model.

**Results:**

Thirty-four adult patients with MSD of supratentorial glioma were enrolled in this retrospective study. The median time to MSD (TTMSD) and overall survival (OS) were 5 months (range: 0–78 months) and 15 months (range: 0.7–85 months), respectively, in the entire cohort. Univariate analysis demonstrated that the patients who had received TMZ therapy had a longer TTMSD than those who did not (mTTMSD: 15 vs. 3 months, log-rank *P* = 0.0004). Furthermore, a protracted duration of salvage chemotherapy of >6 months after MSD was associated with longer OS of the patients with MSD of supratentorial glioma (mOS: 13 vs. 5 months, log-rank *P* = 0.0163) and reduced the death risk by 64.3% (hazard ratio: 0.357, 95% CI: 0.141–0.901, *P* = 0.029) compared with a duration **≤**6 months.

**Conclusion:**

Patients with MSD of supratentorial glioma experienced poor prognosis and adjuvant chemotherapy may delay the occurrence of MSD. The protracted duration of systemic salvage chemotherapy may favor survival after spinal dissemination.

## Introduction

Gliomas are the most common primary tumor in the brain and lead to most brain cancer-related deaths (1, 2). Although the relapse or progression of glioma occurs intracranially in most cases, the metastatic spread of glioma has been described increasingly with prolonged survival from the modest improvements in multimodality therapy of glioma (3–7).

The metastatic spread of glioma is uncommon and typically occurs within the neuroaxis. Among the types of the rare metastatic spread in the neuroaxis, spinal dissemination defined as the involvement of the spinal cord is a less common type of extracranially metastatic spread compared with the spread in intracranial cavity (8, 9).

Considering the low incidence of spinal dissemination of glioma, there is currently no standard of care established and the prognosis remains very poor (10, 11).

Here, we present a retrospective study of over 9 years of experience in our clinic to better understand the clinical course and characteristics of spinal dissemination of supratentorial glioma.

## Methods and Subjects

### Patients and Study Design

Patients with metastatic spinal dissemination (MSD) of supratentorial glioma treated in Beijing Shijitan Hospital between June 2011 and August 2021 were eligible for in this retrospective study, and the demographic, treatment, and survival data were collected for analysis. The pathologic diagnosis of glioma should be histologically confirmed. Spinal dissemination was radiologically diagnosed using enhanced spine magnetic resonance imaging (MRI) by at least two radiologists regardless of whether cerebrospinal fluid (CFS) was positive for tumor cells. Patients with initial glioma of WHO grade I were excluded because of their distinct molecular pathogenesis (12). The other exclusion criteria were age <18 years old, non-supratentorial location of primary glioma, and extracranial dissemination other than spine dissemination after the initial diagnosis of glioma.

The criteria of pathological diagnosis and response assessment were adopted from the 2007 WHO classification and RANO criteria (13–15).

This study was approved by the Institutional Ethical Committee, Beijing Shijitan Hospital. Written informed consent for the research was waived considering the retrospective nature of the study.

### Statistical Analyses

Overall survival (OS) was defined as the interval from the time when spinal dissemination was diagnosed for the first time to death or the latest follow-up. The time to metastatic spinal dissemination (TTMSD) was defined as the time from initial surgery to the point at which spinal dissemination was diagnosed by means of enhanced MRI or CSF examination.

The time to event was estimated using Kaplan–Meier plotting. Univariate analyses were performed using the log-rank test and Cox proportional hazards model. All of the analyses were performed with SPSS (BMI, version 21). *P*-values <0.05 were considered statistically significant.

## Results

### Patient Characteristics

Among the 34 patients included in this analysis, 20 (58.8%) were male and 14 (41.2%) were female, with a median age of 42 years. The histology of the initial tumor was astrocytoma in 28 (82.4%), oligodendroglia in 1 (2.9%), and oligoastrocytoma in 5 (14.7%). At diagnosis, 30 (88.2%) patients underwent surgical resection of the primary tumor (18 GTR, 6 STR, and 6 PR) and 4 patients had biopsy. Following surgery, adjuvant radiotherapy was received in 30 (88.2%) patients, and adjuvant chemotherapy was received in 28 (82.35%) patients [temozolomide (TMZ), 150–200 mg/m^2^/day for the first 5 days of every 28 days] (**Table 1**).

**Table 1:** Patient characteristics at baseline.

Characteristics	Number of cases	Percentage
Age at diagnosis (years), median (range)	37.7 (4.7–66.6)	
Sex		
M	20	58.80%
F	14	41.20%
EoR, primary surgery		
GTR	18	50.00%
STR	6	17.64%
PR	6	14.70%
Biopsy	4	11.76%
WHO grade		
II	9	26.50%
III	10	29.40%
IV	15	44.10%
Origin		
A	28	82.40%
O	1	2.90%
OA	5	14.70%
Histology		
A	8	23.52%
O	0	0.00%
OA	1	2.94%
AA	5	14.70%
AO	1	2.94%
AOA	4	11.76%
GBM	15	44.11%
IDH1/IDH2 status		
Wild type	8	23.53%
Mutated	2	5.88%
Missing	24	70.58%
ATRX status		
Wild type	2	5.88%
Mutated	7	20.50%
Missing	27	79.41%
MGMT promoter status		
Methylated	5	14.70%
Unmethylated	3	8.82%
Missing	26	76.47%
TERT promoter status		
Methylated	3	8.82%
Unmethylated	4	11.76%
Missing	27	79.41%
1p/19q co-deletion		
Yes	1	2.94%
No	4	11.76%
Missing	29	85.29%
Adjuvant RT		
Yes	30	88.20%
No	4	11.80%
Adjuvant TMZ therapy		
Yes	28	82.35%
No	6	17.64%
PFS		
>21 months	9	26.50%
≤21 months	25	73.50%
First relapse location		
Brain	15	44.11%
Spine	14	41.17%
Brain and spine	5	14.70%
Symptom at MSD		
Weakness of lower extremities	13	38.20%
Urinary retention	5	14.70%
Shoulder and back pain	4	11.79%
Headache or seizure	5	14.70%
No symptoms	7	20.58%
Drugs in systemic treatment after MSD		
TMZ	15	44.12%
Platinum	21	61.76%
Nitrosourea	4	11.76%
TKI	3	8.82%
Bevacizumab	3	8.82%
Others	7	20.58%
RT after MSD		
Craniospinal irradiation	3	8.82%
Local irradiation	3	8.82%
No	28	82.40%
IT MTX		
Yes	21	61.76%
No	13	38.24%

M, male; F, female; GTR, gross-total resection; STR, subtotal resection; PR, partial resection; A, astrocytoma; O, oligodendroglioma; OA, oligoastrocytoma; AA, anaplastic astrocytomas; AO, anaplastic oligodendrogliomas; AOA, anaplastic oligoastrocytomas; GBM, glioblastomas; IDH, isocitrate dehydrogenase; ATRX, alpha thalassemia/mental retardation syndrome X-linked; MGMT, O(6)-methylguanine-DNA methyltransferase; TERT, telomerase reverse transcriptase; MSD, metastatic spinal dissemination; RT, radiotherapy; CT, chemotherapy; PFS, progression-free survival; TMZ, temozolomide; TKI, tyrosine kinase inhibitors; IT, intrathecal; MTX, methotrexate.

### Time and Location of MSD

Two patients (one patient with astrocytoma of grade II and one patient with GBM) had primarily a spinal dissemination at the diagnosis of initial glioma and 32 had disseminated relapse on the spine after surgery of the primary tumor. The predominant location of the first relapse was intracranial (15, 44.1%), followed by the spine (14, 41.2 %), and intracranial cavity and the spine dissemination simultaneously (5, 14.7%) (**Table 1**).

Non-surgical therapy was received by 10 patients before diagnosis of metastatic spread. Among these patients, six patients received platinum-based chemotherapy and one patient received semustine treatment after disease relapsed intracranially before spinal dissemination. Spinal dissemination occurred in three patients during the adjuvant therapy of TMZ. The remaining 21 patients have not received non-surgical therapy before diagnosis of metastatic spread except for adjuvant therapy with TMZ.

**Figure 1:**
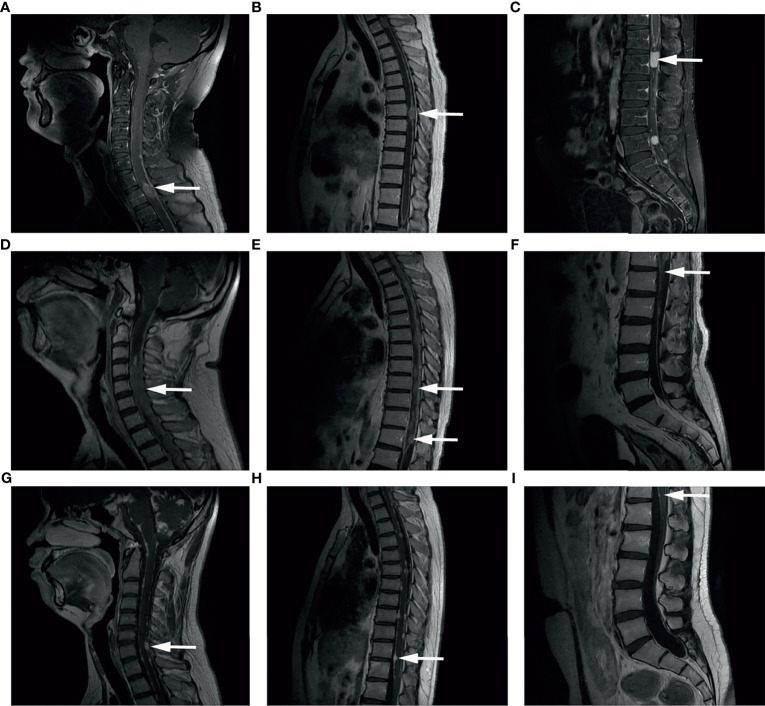
Representative images of Gd-enhanced MRI. Spinal dissemination presented as a hyperintense signal showing as mass or line (arrows) in the spinal canal in T1-weighted MR images. **(A–C)** Spinal Gd-MRI revealed multiple contrast-enhanced mass at the spine in a male aged 34 years old at MSD, who was initially diagnosed as GBM at the left occipital lobe. **(D–F)** Spinal Gd-MRI demonstrated thick linear and clumpy enhanced lesions along the whole spine in a male aged 40 years old at MSD, who was initially diagnosed as anaplastic oligodendroglioma (WHO grade III) at the left frontal lobe. **(G–I)** Spinal Gd-MRI demonstrated small enhanced lesion at the spine in a male aged 17 years old at MSD, who was initially diagnosed as astrocytoma (WHO grade II) at both cerebral hemispheres, cerebellar hemispheres, brainstem surface, ventricles, cisterns, and meninges.

In this study, spinal dissemination was present in any region of the spine and the most common location (67.6%) was the entire spine involvement (**Figure 1**). Six (17.6%) patients showed only cervical lesion, and the remaining five patients had spinal dissemination covering two segments of the spine. Clinical presentation included weakness of lower extremities (for 13 patients), shoulder and back pain (for 4 patients), urinary retention (for 5 patients), and headache or seizure (for 5 patients) which was dependent on the site of disease dissemination. Of note, seven patients had no symptom when spinal dissemination was diagnosed by enhanced MRI.

**Figure 2:**
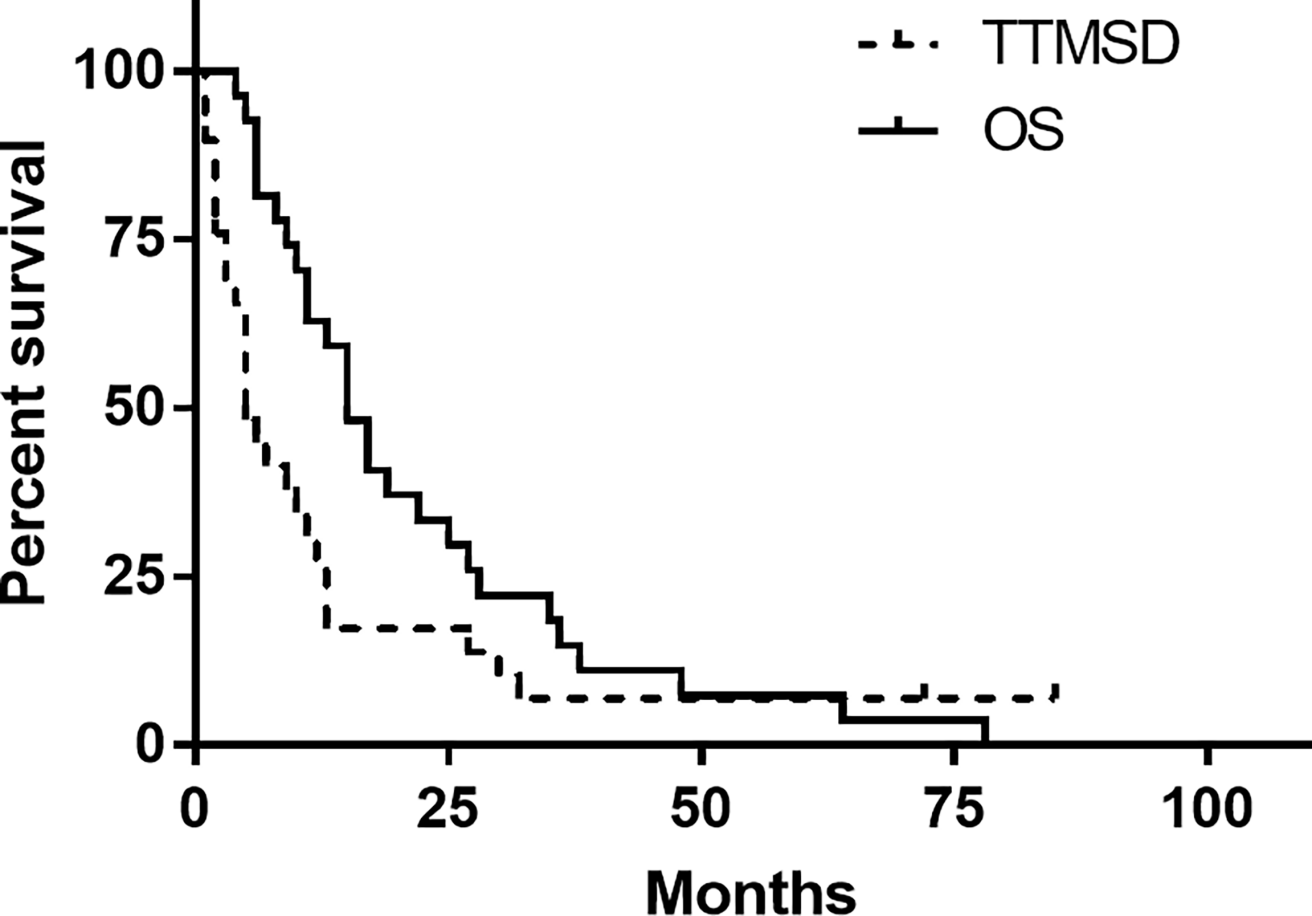
Kaplan–Meier estimated of time to metastatic spinal dissemination (TTMSD) and overall survival (OS).

**Figure 3:**
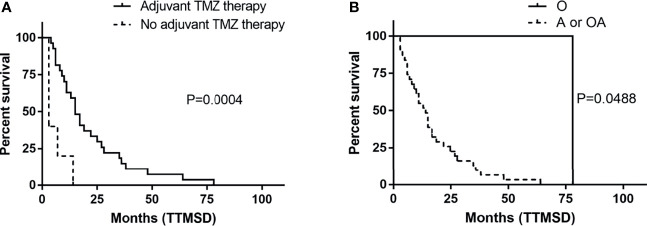
Kaplan–Meier estimated of time to metastatic spinal dissemination (TTMSD) in patients stratified by adjuvant TMZ therapy **(A)** and histologic subtype **(B)**. A, astrocytoma; O, oligodendroglioma; OA, oligoastrocytoma; TMZ, temozolomide.

The TTMSD was estimated using the Kaplan–Meier method. The result indicated that the median TTMSD was 5 months (range: 0–78 months) in the entire cohort (**Figure 2**). When the patients were stratified according to adjuvant chemotherapy, the patients who had received adjuvant TMZ therapy showed longer TTMSD than those who did not (mTTMSD: 15 vs. 3 months, log-rank *P* = 0.0004) (**Figure 3**). In terms of the origin of the tumor, patients with oligodendroglioma appeared to have a longer TTMSD than those with astrocytoma or oligoastrocytoma [mTTMSD: 78 vs. 14 months, log-rank *P* ****=** **0.048, hazard ratio (HR): 0.034, *P* ****=** **0.215] (**Figure 3** and **Table 2**). We observed no significant association between TTMSD and sex, WHO grade, surgery, and adjuvant radiotherapy.

**Table 2:** Univariate analysis of the clinical parameters of the patients with time to metastatic spinal dissemination (TTMSD).

Characteristics	*n*	Median	Univariate analysis		
		TTMSD (months)	HR	95% CI	*P*-value
Age at MSD (years), median (range)	38.6 (6.9–69.6)				
Sex					
M	20	15	1.1012	0.505–2.026	0.974
F	14	13			
Origin					
A/OA	33	14	0.034	0.000–7.125	0.215
O	1	78			
WHO grade					
II	9	20	1.003	0.651–1.545	0.989
III	10	14			
IV	15	10.5			
EoR, primary surgery					
GTR	18	15	1.175	0.857–1.610	0.316
STR	6	16.5			
PR	6	9			
Biopsy	4	15			
Ventricular operative entry					
Yes	16	15	0.82	0.470–1.864	0.85
No	18	13			
Adjuvant TMZ therapy					
Yes	28	15	0.182	0.066–0.502	0.001
No	6	3			
Adjuvant RT					
Yes	30	13.5	0.729	0.253–2.104	0.559
No	4	25.5			

MSD, metastatic spinal dissemination; TTMSD, time to metastatic spinal dissemination; M, male; F, female; A, astrocytoma; O, oligodendroglioma; OA, oligoastrocytoma; EoR, extent of resection; GTR, gross-total resection; STR, subtotal resection; PR, partial resection; TMZ, temozolomide; RT, radiotherapy.

### Treatment After MSD

The majority (76.5%) of the patients had received salvage treatment after spinal dissemination, while four patients received only best supportive care. The drugs utilized in salvage therapy mainly included TMZ (15 patients, 150 mg/m^2^/day, 1 week on and 1 week off), carboplatin (10 patients, AUC = 5, every 4 weeks), and cisplatin (9 patients,75 mg/m^2^ every 4 weeks). Systemic chemotherapy was delivered to the patients with Karnofsky performance status >60% and adequate hematologic, hepatic, and renal function. Six patients received radiotherapy (three for craniospinal irradiation and three for local radiotherapy) after spinal dissemination. Besides systemic chemotherapy, methotrexate (MTX) intrathecal injection was received in 21 patients (**Table 1**).

The duration of systemic salvage therapy was calculated regardless of the various regimens. The median duration of chemotherapy was 5.5 months (1–23 months) for the patients who received at least one cycle of salvage chemotherapy after spinal dissemination. We found that the duration >6 months was observed in seven patients, while five patients received systemic therapy for >10 months after spinal dissemination.

### Overall Survival

At the time of this report, 32 (94.1%) of the 34 patients had died. The follow-up time for the 2 remaining patients still alive is 72 and 85 months, respectively.

**Figure 4:**
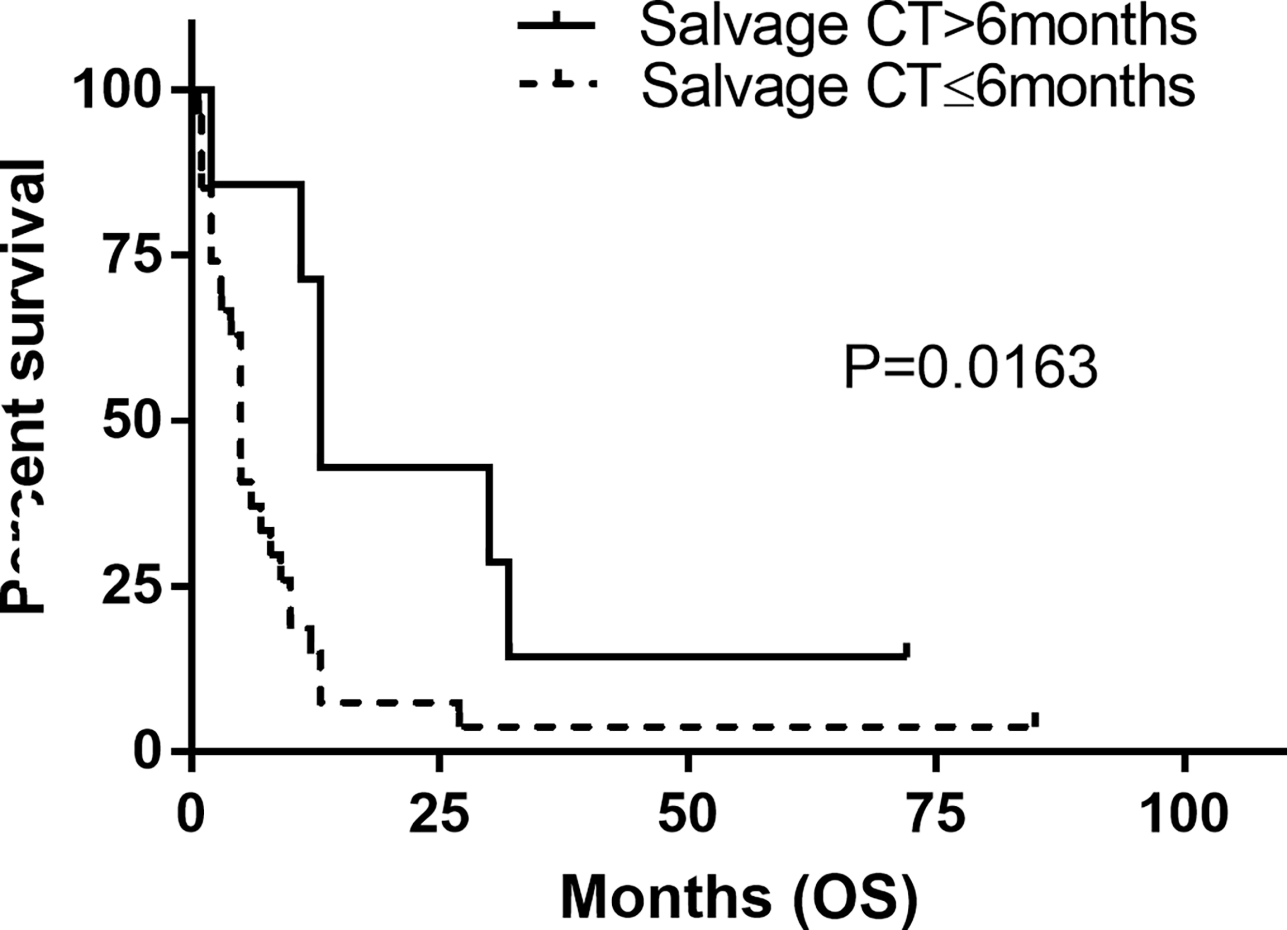
Kaplan–Meier estimated of overall survival (OS) in patients stratified by duration of systemic salvage therapy. Salvage CT, salvage chemotherapy.

The estimated median OS by Kaplan–Meier plotting was 15 months (range: 0.7–85months) in this study (**Figure 2**). On univariate analysis, a protracted duration of salvage chemotherapy of >6 months after MSD was associated with longer OS of the patients with MSD of supratentorial glioma (mOS: 13 vs. 5 months) and reduced the death risk by 64.3% (HR 0.357, 95% CI: 0.141–0.901, *P* ****=** **0.029) compared to a duration of <6 months (**Table 3** and **Figure 4**). However, no statistical significance was found between the groups concerning sex, histological classification, surgery and postoperative therapy, age of dissemination, whether there was dissemination for first recurrence, whether therapy was received after dissemination, the range of dissemination, or progression-free survival.

**Table 3:** Univariate analysis of the clinical parameters of the patients with overall survival (OS).

Characteristics	*n*	Median OS (months)	Univariate analysis		
			HR	95% CI	*P*-value
Age at MSD (years), median (range)	38.6 (6.9–69.6)				
Age at MSD					
<60	33	6	1.617	0.214–12.206	0.641
≥60	1	5			
Sex					
M	20	5	0.825	0.408–1.670	0.593
F	14	9			
Origin					
A/OA	33	6	1.617	0.214–12.206	0.641
O	1	5			
WHO grade					
II	9	10	1.073	0.693–1.661	0.753
III	10	6.5			
IV	15	5			
EoR, primary surgery					
GTR	18	5	0.865	0.623–1.201	0.387
STR	6	10			
PR	6	7			
Biopsy	4	17.5			
Ventricular operative entry					
Yes	16	7.5	0.908	0.451–1.828	0.787
No	18	5			
Adjuvant TMZ therapy					
Yes	28	5	1.428	0.548–3.719	0.466
No	6	9			
Adjuvant RT					
Yes	30	5.5	1.588	0.488–5.255	0.449
No	4	8			
PFS					
>21	9	9	0.618	0.274-1.395	0.247
≤21	25	5			
First relapse location					
Brain	15	5	0.740	0.457–1.198	0.220
Spine	14	5			
Brain and spine	5	11			
Region of MSD					
Whole spine (C+T+L)	23	5	1.265	0.788–2.029	0.331
Two segments involved (C+T or T+L or C+L)	5	8			
One segment involved	6	7.5			
Salvage CT					
Yes	26	6.5	1.539	0.612–3.865	0.359
No	8	3.5			
Duration of salvage CT					
>6 months	7	13	0.357	0.141–0.901	0.029
≤6 months	27	5			
Salvage RT					
Yes	6	5	1.539	0.612–3.865	0.359
No	28	6.5			
IT MTX					
Yes	21	5	0.91	0.442–1.872	0.798
No	13	7			

OS, overall survival; MSD, metastatic spinal dissemination; M, male; F, female; A, astrocytoma; O, oligodendroglioma; OA, oligoastrocytoma; EoR, extent of resection; GTR, gross-total resection; STR, subtotal resection; PR, partial resection; adjuvant RT, adjuvant radiotherapy; TMZ, temozolomide; salvage CT, salvage chemotherapy; salvage RT, salvage radiotherapy; PFS, progression-free survival; C, cervical; T, thoracic; L, lumbar; IT, intrathecal, MTX, methotrexate.

## Discussion

The spinal dissemination of supratentorial glioma is rare and represents a late event of the disease course (16, 17). The mechanism underlying the metastatic dissemination and prognostic factors is unclear. In this study, we found that adjuvant chemotherapy may delay the occurrence of MDS and that a protracted duration of salvage chemotherapy after MSD significantly prolonged the OS of the patients with MSD of supratentorial glioma.

Spinal dissemination was first reported and is more common in glioblastoma multiforme (GBM), a grade IV tumor with the most aggressive characteristics (18, 19). However, according to our own clinical experience and series of reports, spinal dissemination may also occur in patients with low-grade glioma (20, 21). In this study, on univariate analysis, adjuvant chemotherapy was identified to be associated with longer TTMSD indicating that MSD occurred later in patients with adjuvant chemotherapy than those without. The results suggest that adjuvant therapy is important and should be performed considering the WHO grade and other risk factors according to the NCCN guidelines.

In this study, patients with MSD of supratentorial glioma of WHO grades II (9 patients), III (10 patients), and IV (15 patients) were included, and the median overall survival after spinal dissemination was 15 months for all the patients. This rather long survival time for patients with MSD possibly contributes to a better prognosis of patients with low-grade glioma (LGG) in this cohort as well as clinical benefit from salvage treatment after MSD. It is worth noting that the span of survival range was rather wide suggesting that survival time differed between individuals, although the underlying mechanism remains unclear.

When the outcomes of patients with different WHO grades of initial tumor were compared, we found that the median survival time after MSD in patients with tumor of grade II (10 months) was longer than that in patients with tumor of grade III (6.5 months) and grade IV (5 months) in value, although no significant difference was found by statistical analysis. The limited sample size maybe the possible reason. Nevertheless, the results suggested that, on the one hand, the prognosis of patients with MSD of supratentorial glioma is poor as soon as the dissemination occurs regardless of the initial historical grade, and on the other hand, the prognosis of patients with MSD seemed to be better in patients with primary LGG than in patients with primary HGG. The median survival after MSD for patients with primary malignant glioma in this study is consistent with previous literature (22).

We have to address the issue that adults with low-grade astrocytomas sometimes progress to high-grade diseases (23). Among the nine patients with MSD after primary astrocytoma of grade II enrolled in this study, one patient received resection when disease relapsed and HGG was confirmed histologically which reflected the fact of evolution of LGG. However, it is difficult to evaluate the contribution of evolution or transformation of LGG in the process of MSD occurrence and development due to so limited data in this study.

As far as we know, there is no standard of care established for MSD of glioma. In this study, multiple regimens or drugs were used in the salvage systemic treatment of MSD. The specified regimen or drug failed to bring clinical benefit on the overall survival of patients considering the limited sample size and unbalance distribution in each group of treatment. However, we found that a longer duration of systemic chemotherapy favored survival after spinal dissemination. This result indicated that clinical benefit could be conferred by systemic chemotherapy after spinal dissemination for partial patients.

Among the patients who had received systemic treatment after spinal dissemination, most of them received TMZ or platinum-based chemotherapy. Generally, patients who received chemotherapy showed better clinical condition compared with patients who did not. We could not exclude the possibility that patients who received chemotherapy had longer survival might be the cause or consequence of clinical conditions. Thus, how to screen specific patients who may gain benefit from salvage chemotherapy should be addressed in a prospective study with a larger sample size.

It has been reported that male, age, and ventricular operative entry are risk factors for spinal dissemination of glioma (6, 24). Spinal dissemination was reported to more frequently occur in young patients (25) because younger patients tend to have longer survival time. Meanwhile, patients with high-grade glioma who are >65 years old only survive 7–9 months (26). In this study series, the average age at dissemination was 38.9 years old and 16 patients had experienced possible opening of the ventricular system.

This study has several limitations. First, the retrospective nature of this study may be a source of potential bias and may exclude meaningful comparison and conclusion to some extent. Second, the limited sample may also contribute to the bias. Third, the lack of molecular characteristic information for a considerable number of patients and multiple modality or drugs used in salvage treatment after MSD make it hard to compare and figure out factors related to the prognosis of patients with MSD.

Considering the preliminary result from this retrospective study with limited sample size, well-designed prospective studies with an expanded sample will be performed in the future.

## Conclusion

In conclusion, the prognosis of patients with MSD of supratentorial glioma is considered poor as soon as dissemination occurs regardless of the initial historical grade, and adjuvant chemotherapy may delay the occurrence of MSD. Moreover, a protracted duration of systemic salvage chemotherapy may favor survival after spinal dissemination.

## Data Availability Statement

The raw data supporting the conclusions of this article will be made available by the authors, without undue reservation.

## Ethics Statement

The studies involving human participants were reviewed and approved by the Institutional Ethics Committee of Beijing Shijitan Hospital of Capital Medical University [No. sjtkyll-lx-2021(64)]. Written informed consent to participate in this study was provided by the legal guardian/next of kin of the participants.

## Author Contributions

Conception and design: JC and HH. Provision of study materials and patients: JC and YZ. Collection and assembly of data: QS, FY, and JC. Data analysis and interpretation: FY and HH. Manuscript writing: all authors. Final approval of the manuscript: all authors.

## Funding

This work was funded by the National Natural Science Foundation of China (grant number 81572799).

## Conflict of Interest

The authors declare that the research was conducted in the absence of any commercial or financial relationships that could be construed as a potential conflict of interest.

## Publisher’s Note

All claims expressed in this article are solely those of the authors and do not necessarily represent those of their affiliated organizations, or those of the publisher, the editors and the reviewers. Any product that may be evaluated in this article, or claim that may be made by its manufacturer, is not guaranteed or endorsed by the publisher.
